# Trends in Hip Fracture Incidence, Length of Hospital Stay, and 30-Day Mortality in Sweden from 1998–2017: A Nationwide Cohort Study

**DOI:** 10.1007/s00223-022-00954-4

**Published:** 2022-02-15

**Authors:** Peter Nordström, Jonathan Bergman, Marcel Ballin, Anna Nordström

**Affiliations:** 1 Department of Community Medicine and Rehabilitation, Unit of Geriatric Medicine, Umeå University, 90187 Umeå, Sweden; 2 Department of Public Health and Clinical Medicine, Section of Sustainable Health, Umeå University, Umeå, Sweden; 3 School of Sport Sciences, UiT the Arctic University of Norway, Tromsø, Norway

**Keywords:** Fracture, Incidence, Mortality, Epidemiology, Risk factors

## Abstract

In this nationwide cohort study, we investigated trends in hip fracture incidence, hospital length of stay (LOS), and 30-day mortality after admission in Sweden. The cohort included all individuals in Sweden aged ≥ 50 years with a first hip fracture diagnosis during 1998–2017 (*N* = 313,761). The outcomes were hip fracture incidence and 30-day mortality. The results showed that the incidence of hip fracture decreased from 79.2 to 46.7/10,000 population in women and from 35.7 to 26.5/10,000 population in men. In contrast, 30-day mortality increased from 4.3% to 6.2% in women (*P* < 0.001) and from 8.4% to 11.1% in men (*P* < 0.001). Strong risk factors (*P* < 1 × 10^–25^ for all) for 30-day mortality were older age, male sex, and shorter LOS. From 1998 to 2017, LOS decreased by on average 4 days and was shorter in both male and female aged ≥ 90 years compared to those aged 80–89 (*P* < 0.001 for comparisons). In conclusion, despite population aging, hip fracture incidence in Sweden has decreased over the last 20 years. However, short-term mortality has increased, which to some extent may partly be explained by the fact that LOS has decreased without being adapted to important risk factors.

**Supplementary Information:**

The online version contains supplementary material available at 10.1007/s00223-022-00954-4.

## Introduction

In 2000, an estimated nine million fragility fractures occurred worldwide [1], with adverse consequences including chronic pain [2], disability, and premature death [3, 4]. The hip fracture is the most devastating type of fracture both for the individual and the society [5], with nationwide studies and meta-analyses showing that about 25% of hip fracture patients die within a year of the event [4, 6, 7]. In addition, among those that survive, recovery to pre-fracture functional level is often unsuccessful [8].

From the 1950s to the mid-1990s, the incidence of hip fracture increased in many countries [9–12], a trend that coincided with population aging [13]. Despite continued population aging, there are studies suggesting that the incidence of hip fractures has leveled off [14, 15], or even decreased according to the most recent studies [16–18], although the cause of this is uncertain [17, 19]. There also seems to be substantial variation when comparing these trends in low-, middle-, and high-income countries [20]. Given these trends and variations, contemporary nationwide studies spanning over decades would be of interest, exploring also possible reasons for a decreasing incidence.

In addition to the public health burden of hip fractures, these fractures are considered a good measure of the quality of healthcare [21], where inpatient length of stay (LOS) may not only be adapted to patient demands, but also influenced by economic constraints in health care. A previous study from Sweden reported declining hip fracture incidence during the last two decades but lack of improvement in mortality rates [18]. However, neither time trends in LOS nor the influence of LOS on short-term mortality were investigated [18]. In an earlier study, we showed that in hip fracture patients, shorter LOS was associated with increased risk of death after discharge [6]. These results were confirmed in a recent study investigating hip fracture patients with dementia [22]. By implication, it could be hypothesized that efforts to reduce LOS related to economic constraints may have a negative influence on absolute short-term mortality over time. In this study, we, therefore, investigated the trends in hip fracture incidence, LOS, and 30-day mortality after hospitalization for hip fracture in the Swedish population during 1998–2017.

## Material and Methods

### Study Population

For the present study, we included all individuals in Sweden with a first hip fracture at an age of at least 50 years in 1998 until 2017. Hip fractures were tracked using the Swedish National Patient Register (NPR), controlled by the National Board of Health and Welfare (www.socialstyrelsen.se), using the International Classification of Diseases, 10th revision, (ICD-10) codes S720 (fractures of the femoral neck), S721 (fractures of the trochanter) and S722 (subtrochanteric hip fractures). For the fractures of the femoral neck and trochanter, we also obtained information about the type of operation (procedure codes NFB [operation with prosthesis], NFJ [operation with internal fixation]). Diagnoses in inpatient health care have been collected in the NPR since the late 1960s, with complete coverage since 1987. To estimate the yearly incidence of hip fractures, the size of the Swedish population above 50 years of age in 1998–2017 was obtained from Statistics Sweden [23].

### Covariates

Covariates were selected based on known associations with hip fractures or death. Diagnoses were obtained from the NPR using the following ICD-10 codes: I21 (myocardial infarction), I61-I64 (stroke), I20 (angina pectoris), E10, E11 (diabetes mellitus), N17-N19 (kidney failure), J43, J44 (chronic obstructive pulmonary disease, COPD), F10 (alcohol dependency), F32-F33 (depression), C (cancer), and F00-F03 (dementia). We included only diagnoses set within one year prior to the fracture because the NPR switched to ICD-10 codes in 1997, so only one year of ICD-10 codes was available for hip fracture patients in 1998 (the first year of the study).

Socioeconomic data, including disposable income, early retirement pension, and marital status (married, never married, divorced, or widowed), were obtained from Statistics Sweden for each patient in the year before the fracture occurred. The data were missing in 0.1% of the patients. The Swedish Prescribed Drug Register was used to collect data on all prescriptions for bone-specific agents (anatomical therapeutic classification code, M05B) dispensed at pharmacies in Sweden since July 2005. Specifically, we tracked use of bone-specific agents within the past year in patients that sustained hip fractures in 2007 and later. Finally, hospital LOS was estimated from the NPR, where the date of admission and discharge were recorded. If a patient was transferred from one ward to another on the same date, the separate LOS were summed. The present study was approved by the Swedish Ethical Review Authority, number 2019-04765, and by the National Board of Health and Welfare, and by Statistics Sweden.

### Statistics

Data in the present study were presented as means and standard deviations, unless stated otherwise. Differences between two groups were investigated using a Student’s t-test for independent samples. The mean change in hip fracture incidence per year was estimated using linear regression with autoregressive moving average (ARMA) errors, to account for possible dependency of errors. To estimate mean percentage change in incidence, we used the same model but applied the natural logarithmic transformation to hip fracture incidence (the outcome). The order of the ARMA model was selected as the minimum order for which there was no significant autocorrelation or partial autocorrelation. Separate models were run for men and women and each age group.

The risk of death within 30 days of admission was investigated using Cox regression. LOS was entered as a time-varying variable (value 1 on the day of hospitalization, 2 on the second day of hospitalization, and so on, until the day of discharge, after which it was constant). The Cox model was adjusted for age, sex, income, early retirement pension, marital status (4 categories), type of fracture (3 categories), type of operation (6 categories) and 10 different diagnoses. In a sensitivity analysis, individuals were included with a LOS of 10 days or less and alive at discharge. The outcome in this analysis was death from day 11 to 30 after admission and LOS was included as a non-time dependent covariate. Data on mortality were obtained from the Swedish Cause of Death register, which is complete since 1952. [24] All analyses were performed using Stata MP, version 16.1 for Mac (StataCorp, College Station, TX 77845, USA) and RStudio (R, version 4.0.3). P values of less than 0.05 and 95% confidence intervals (CI) not including one was considered significant.

## Results

### Study Cohort

From 1998 to 2017, Sweden’s population aged 50 or older increased from 3.13 million (33.5% of the total population) to 3.85 million (38.4% of the total population) individuals. During this period, a total of 313,761 individuals were diagnosed with a first hip fracture. Some of the variables describing baseline characteristics of the hip fracture patients changed between 1998 and 2017 (Table 1, Supplementary Table 1). Inpatient LOS decreased from a mean of about 14 days in 1998 to 10 days in 2017 (*P* < 0.001). In terms of operation techniques, there was a substantial increase in patients operated with prosthesis after a femoral neck fracture. The proportions of patients with a recent myocardial infarction, stroke, or angina pectoris at the time of the hip fracture decreased during follow-up. In 2007, 1.6% of the patients were prescribed a bone-specific drug in the year before the fracture compared to 1.2% in 2017.

**Table 1:** Basal characteristics of the study cohort in 1998 (*N* = 18,534) and in 2017 (*N* = 14,188)

Variables	1998	2017
*Age, years*	81.3 ± 9.0	81.9 ± 9.8
*Female sex (%)*	72	65
*Income (1000 Swedish krona)*	95 ± 60	190 ± 389
*Early retirement pension (%)*	3.1	2.7
*Missing, %*	0.1	0.1
*Marital status, %*		
-Widow/widower	48.6	38.9
-Married	30.3	33.6
-Not married	11.0	10.6
-Divorced	10.0	16.7
*Missing, %*	0.1	0.1
*Length of stay, days (mean* ± *SD)*	14.8 ± 14.4	10.2 ± 7.5
*Type of fracture and operation*		
*Collum femoris fracture (%)*		
-Operated with prosthesis	4.4	32.6
-Operated with nails	18.6	7.4
-Operated with screws	16.5	6.8
-Operated with other technique/unknown	14.9	7.4
*Trochanteric fracture (%)*		
-Operated with intermedullary nail	1.4	19.3
-Operated with combined technique	26.6	12.8
-Operated with other technique/unknown	10.6	6.1
*Subtrochanteric fracture (%)*	7.0	7.6
*Cause of fracture (%)*		
-Snow or ice	3.6	3.3
-Inside	71.3	57.1
*Debut of diagnoses the last year, %*		
-Myocardial infarction	1.2	0.7
-Stroke	2.9	1.6
-Angina pectoris	2.2	0.4
-Diabetes	2.7	1.3
-Kidney failure	0.6	1.5
-Obstructive pulmonary disease	1.0	0.7
-Alcohol dependency	3.0	2.3
-Depression	1.1	0.5
-Cancer	1.8	2.2
- Dementia	2.8	2.4

### Incidence and Total Number of Hip Fractures, 1998–2017

**Fig. 1:**
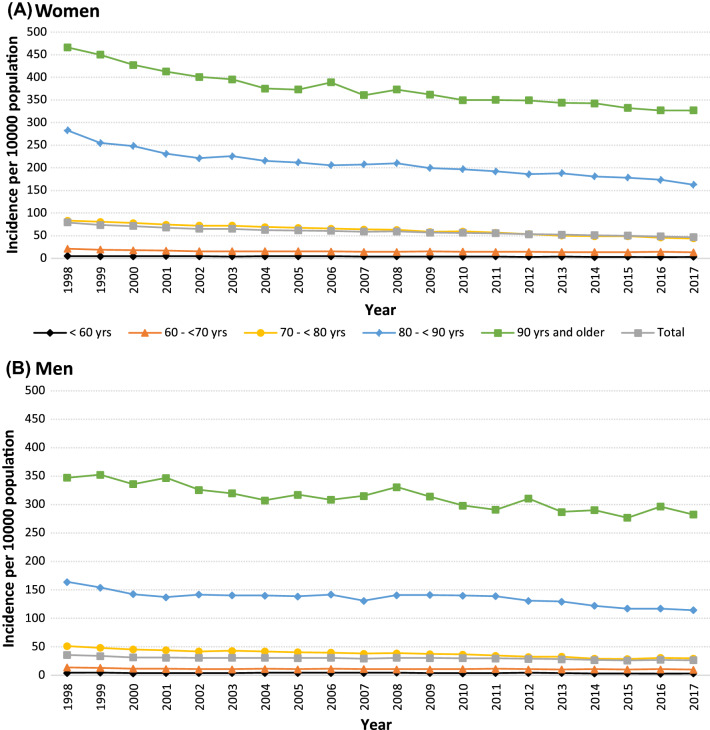
Incidence of hip fractures from 1998 to 2017 in Swedish women (**A**) and in men (**B**) in total and according to age groups

The incidence of hip fractures decreased from 79.2 to 46.7 per 10,000 population in women and from 35.7 to 26.5 per 10,000 population in men, equal to a mean decrease of 1.4 (95% CI, 1.6–1.3; *P* < 0.001) and 0.4 (95% CI, 0.5–0.3; *P* < 0.001) cases per year and 10,000 population, in women and men, respectively (Fig. 1). A significant relative risk reduction was observed in all age groups, but the absolute risk reduction was higher in older age groups (Table 2). Furthermore, the total number of hip fractures decreased between 1998 and 2017, from 13,340 to 9266 cases in women, and from 5194 to 4922 fractures in men (Supplementary Fig. S1).

**Table 2:** Mean change in yearly hip fracture incidence by age and sex

	Mean change in annual cases/10,000 population (95% CI)		Mean percentage change/year (95% CI)	
Age group	Men	Women	Men	Women
<60	−0.1 (−0.1, −0.0)	−0.1 (−0.1, −0.1)	−1.5 (−2.3, −0.7)	−2.4 (−2.9, −1.9)
60–69	−0.1 (−0.2, −0.1)	−0.3 (−0.4, −0.2)	−0.9 (−1.3, −0.6)	−1.9 (−2.5, −1.4)
70–79	−1.1 (−1.2, −1.0)	−2.0 (−2.1, −1.9)	−2.8 (−3.0, −2.5)	−3.2 (−3.4, −3.0)
80–89	−1.9 (−2.4, −1.3)	−4.8 (−5.4, −4.1)	−1.4 (−1.8, −1.0)	−2.2 (−2.5, −2.0)
≥90	−3.4 (−4.1, −2.6)	−6.5 (−7.7, −5.3)	−1.1 (−1.3, −0.8)	−1.7 (−1.9, −1.4)

### A 30-Day Mortality After Hip Fracture, 1998–2017

**Fig. 2:**
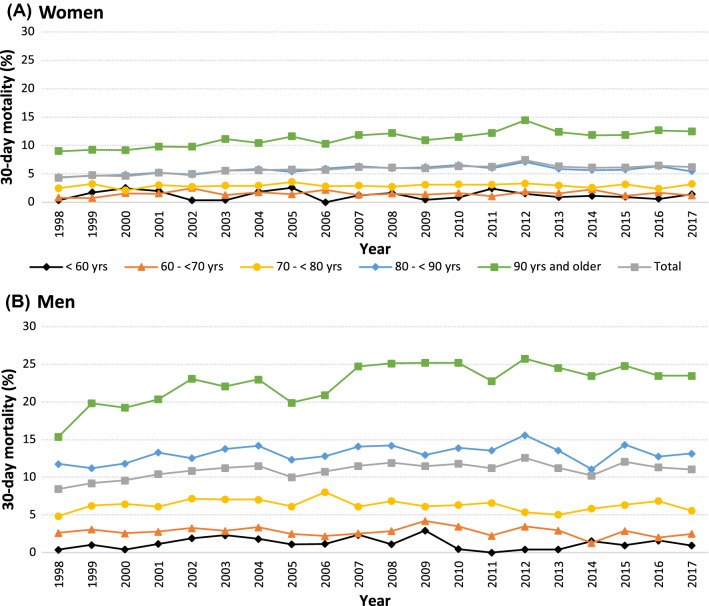
Risk of death within 30 days of admission from 1998 to 2017 in Swedish women (**A**) and in men (**B**) in total and according to age groups

Between 1998 and 2017, 30-day mortality after hip fracture increased from 4.3% to 6.2% in women and from 8.4% to 11.1% in men (Fig. 2). In unadjusted analyses, this was equal to a mean increase of 1.9% (Hazard ratio, [HR], 1.019, 95% CI, 1.016–1.022, *P* < 0.001) and 1.1% per year (HR, 1.011, 95% CI, 1.007–1.014 *P* < 0.001), in women and men, respectively. In men aged 90 and above, the absolute risk of 30-day mortality increased by 8.1% and the relative risk increased by 53%, from 1998 to 2017, which was higher than for any other age group of women or men (Fig. 2). In the total cohort, the strongest risk factors (*P* < 1 × 10^–25^ for all) for 30-day mortality in the model included age (HR, 1.08, 95% CI, 1.08–1.09, per year higher age), male sex (HR, 2.34, 95% CI, 2.27–2.40), and LOS (HR, 0.96, 95% CI, 0.96–0.96 per day longer LOS). Other strong risk factors included type of fracture and operation, and diagnosis of kidney failure (HR, 1.80, 95% CI, 1.63–1.98), myocardial infarction (HR, 1.70, 95% CI, 1.55–1.86), COPD (HR, 2.05, 95% CI, 1.84–2.28) and cancer (HR, 1.63, 95% CI, 1.52–1.76) within one year prior to the hip fracture.

### LOS After Hip Fracture

**Fig. 3:**
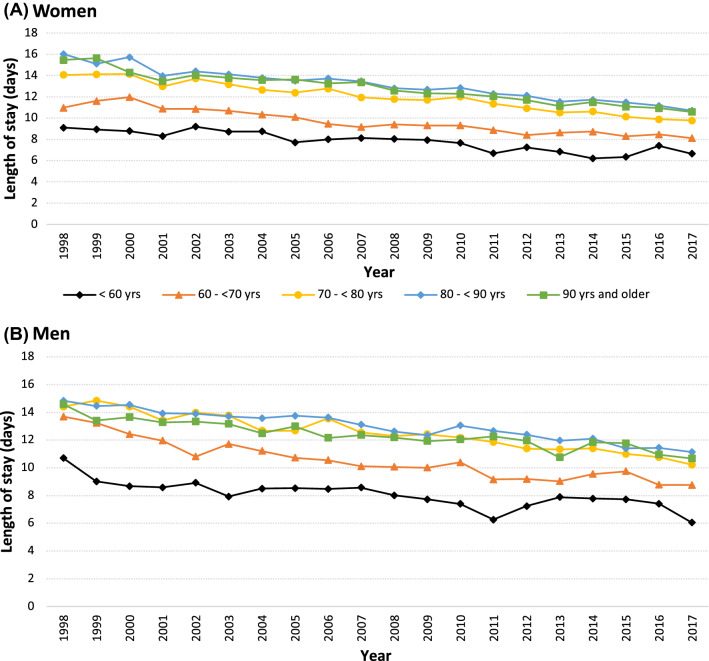
Inpatient length of stay in female hip fracture patients (**A**) and in male hip fracture patients (**B**) from 1998 to 2017 according to age groups

LOS was not adapted to risk factors for 30-day mortality, such as male sex or older age (Fig. 3). Specifically, mean LOS was similar in men and women during the study period, and it was longer in patients aged 80–89 years than in patients aged 90 years or above (13.0 ± 11.0 vs. 12.3 ± 10.2 days in men, *P* < 0.001 for comparison; 13.3 ± 11.3 days vs. 12.8 ± 10.3 days in women, *P* < 0.001 for comparison). In comparison, 30-day mortality was lower in patients aged 80–89 years than in patients aged 90 years or above (13.2% vs. 22.9% in men, *P* < 0.001 for comparison; 5.6% vs. 11.3% in women, P < 0.001 for comparison). LOS was rather similar in patients with diagnoses that showed that the strongest association with 30-day mortality. Thus, in unadjusted analyses, mean LOS by comorbidity was 13.3 ± 11.2 days (kidney failure), 13.6 ± 11.4 days (myocardial infarction), 13.8 ± 12.2 days (stroke) and 13.3 ± 11.9 days (COPD), compared to 12.5 ± 10.7 days in the total cohort.

In a sensitivity analysis, only individuals with a LOS of 10 days or less were included that were alive at discharge (*N* = 159,924). Outcome included death from day 11 to days 30 after admission, and LOS was included as an explanatory variable (non-time dependent) together with the other covariates. In the model including all covariates, the strongest risk factors (*P* < 1 × 10^–25^ for all) for 30-day mortality included LOS (HR, 0.91, 95% CI, 0.91–0.92, per day longer LOS), age (HR, 1.08, 95% CI, 1.07–1.08, per day higher age), and female sex (HR, 0.46, 95% CI, 0.44–0.49).

## Discussion

This nationwide study showed that both the incidence and the total number of hip fractures decreased in Sweden between 1998 and 2017, despite a concurrent aging of the population. In contrast, 30-day mortality after admission increased in both women and men. Strong risk factors for higher mortality included older age, male sex, and LOS which decreased with 4 days during the study period. Possibly, the fact that LOS was not adapted to these risk factors may have influenced the increase in short-term mortality that was observed during the study period.

The result of the present study contradicts projections which suggest that hip fractures will become more common in the future [13, 14], but are supported by recent studies from high-income countries [16–18, 25]. In our population, there was no sign for this decreasing trend to level off, which is of great interest with respect to other populations around the world where the number of older people is also increasing. Any reasons for the declining number of fractures in our nationwide cohort would be of interest to evaluate. We found no evidence of an increased use of bone-specific agents during the last decade in Sweden, a pattern which appears similar to that observed in other countries [26]. It could also be argued that the decreasing incidence of hip fractures may relate to climate change, resulting in less snow and ice. However, less than 4% of all hip fractures were diagnosed as due to snow and ice, suggesting that also this explanation is unlikely. Other factors, such a decreased use of tobacco, [25, 27] unexpected potential protective effects of commonly used drugs [28, 29], or a lower burden of concomitant disease, all of which reflect a healthier aging, may have influenced the hip fracture incidence in our study. Indeed, according to annual investigations by the Public Health Agency of Sweden, self-reported smoking decreased from 13% to 7% in the Swedish population between 2006 and 2020 [30]. In our cohort there was also a decreasing prevalence of concomitant cardiovascular disease during follow-up, further supporting a healthier aging.

During follow-up, 30-day mortality increased in both men and women. This observation stands in contrast to the decreased mortality seen in other large non-communicable diseases, such as myocardial infarction [31]. The increased 30-day mortality also stands in contrast to a study from the US, where age- and risk-adjusted mortality after hip fracture was slightly reduced between 1985 and 2005 [12], Although these data are older, it is also of interest that LOS was reduced to half during the study period, and that another more recent study from the US found no association between shorter LOS and mortality in hip fracture patients [32]. Furthermore, in a study from the UK, 30-day mortality after hip fracture decreased from 12 to 6% between 2006 and 2012 [33]. Thus, the opposite trend observed in Sweden is clearly a cause of concern. In a recent study, using a similar cohort from Sweden, mortality remained rather constant, and was somewhat improved during the years of follow-up when adjusted for Charlson comorbidity index [18]. The different covariates used compared to in our study may explain the somewhat different results. In our nationwide cohort, higher 30-day mortality was predominantly explained by higher age, male sex, and a shorter inpatient LOS during follow-up. Because hip fracture patients are generally frail, have multimorbidity, and are at high risk of complications and ultimately death [4, 34, 35], it seems rather clear that LOS should be adapted to each patient’s individual risk factors. However, we noted that LOS was similar in women and men, and shorter in patients aged at least 90 years of age, compared to those aged 80–89 years of age. If the relationship is causal, it is possible that the shorter LOS may have influenced mortality during the study period, which was highest in men aged 90 and above, and also increased most in this frail patient group during the study period.

The present study has limitations that should be considered. First, although hospitals are staffed by highly experienced and educated personnel, the observational design of this study means that we cannot precisely determine to which extent a longer LOS would reduce 30-day mortality, for example in frail men aged 90 and above. Second, we lacked certain covariates of importance, such as an adequate measure of the patient’s health status at admission, which may have changed during the study period, as well as data on type of hospital and post-anesthesia care unit LOS, which may influence the 30-day mortality estimates [36, 37]. Third, there were some interesting observations with respect to trends in operation techniques, such as a shift toward use of intramedullary nails for intertrochanteric fractures and toward prosthesis for femoral neck fractures. However, we were unable to investigate associations between these trends and the risk of short-term mortality or disability due to the lack of certain important covariates such as patient’s health status at admission and follow-up data on mobility. This remains an area for future investigation. Fourth, reliable data on cause-specific deaths are not available from Swedish national registers. Yet, for the outcome 30-day mortality after a hip fracture, the operation and fracture are generally regarded as strong contributing causes of death, with complications including infection and cardiovascular disease [38, 39]. The strengths of the study include the long study duration of 20 years, the use of national registers, which ensured complete coverage of all hip fractures in Sweden and no loss to follow-up for 30-day mortality, altogether increasing the external and internal validity of the study results.

In summary, during the past 20 years, both the number and incidence of hip fractures in Sweden has decreased, despite the aging of the population. Possibly, this trend may partly be explained by favorable changes in the prevalence of smoking and concomitant disease in older people. In contrast, 30-day mortality increased during the study period, where higher mortality was predominantly associated with male sex, higher age, and shorter LOS, the latter which also decreased during the study period. With the limitation of being an observational study, these results indicate that shortening LOS may potentially have a detrimental impact on the risk of subsequent complications and death in frail patients with hip fracture.

## Supplementary Information

Below is the link to the electronic supplementary material.

Supplementary file1—Supplementary Fig. S1 Total number of hip fractures from 1998-2017 in Swedish women (A) and in men (B) in total and according to age groups. (PDF 58 kb)

Supplementary file2 (DOCX 24 kb)
