# Supplementary material for: Trends in Hip Fracture Incidence, Length of Hospital Stay, and 30-Day Mortality in Sweden from 1998–2017: A Nationwide Cohort Study
Source: Calcif Tissue Int. 2022 Feb 15;111(1):21–8. doi: 10.1007/s00223-022-00954-4 (PMC9232476; doi:10.1007/s00223-022-00954-4)

Supplementary Figure S1. Total number of hip fractures from 1998-2017 in Swedish women (A) and in men (B) in total and according to age groups.

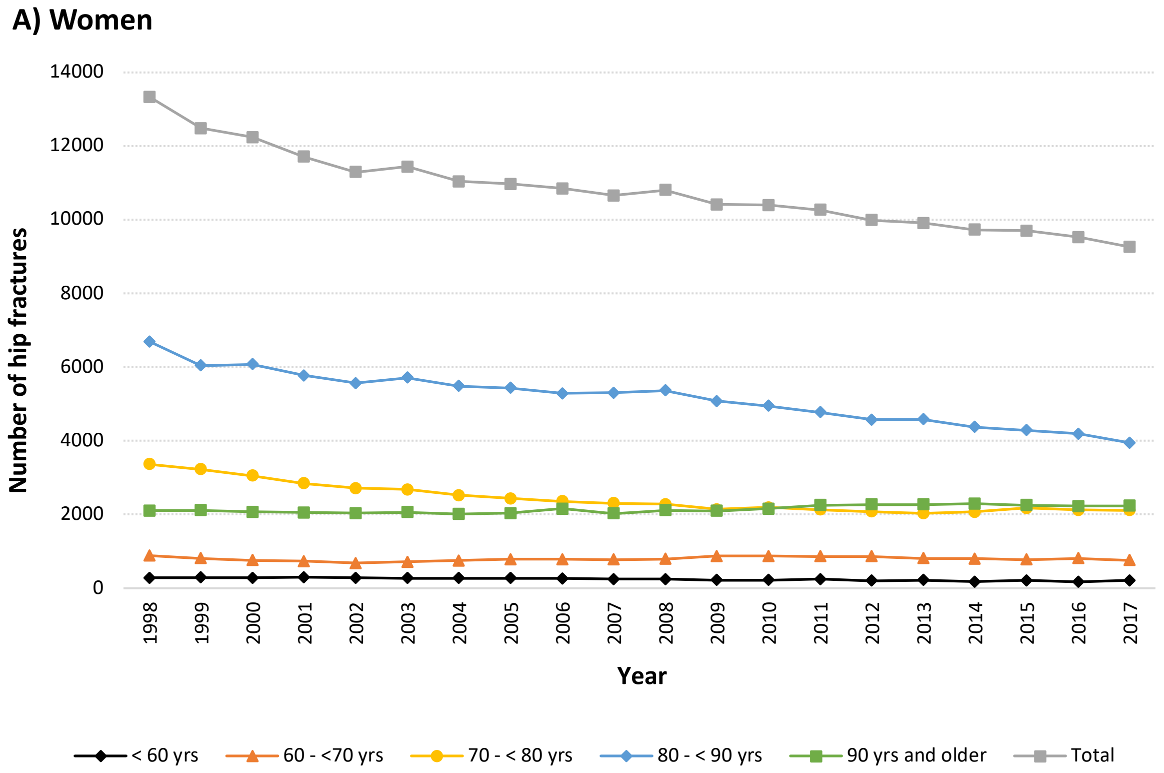

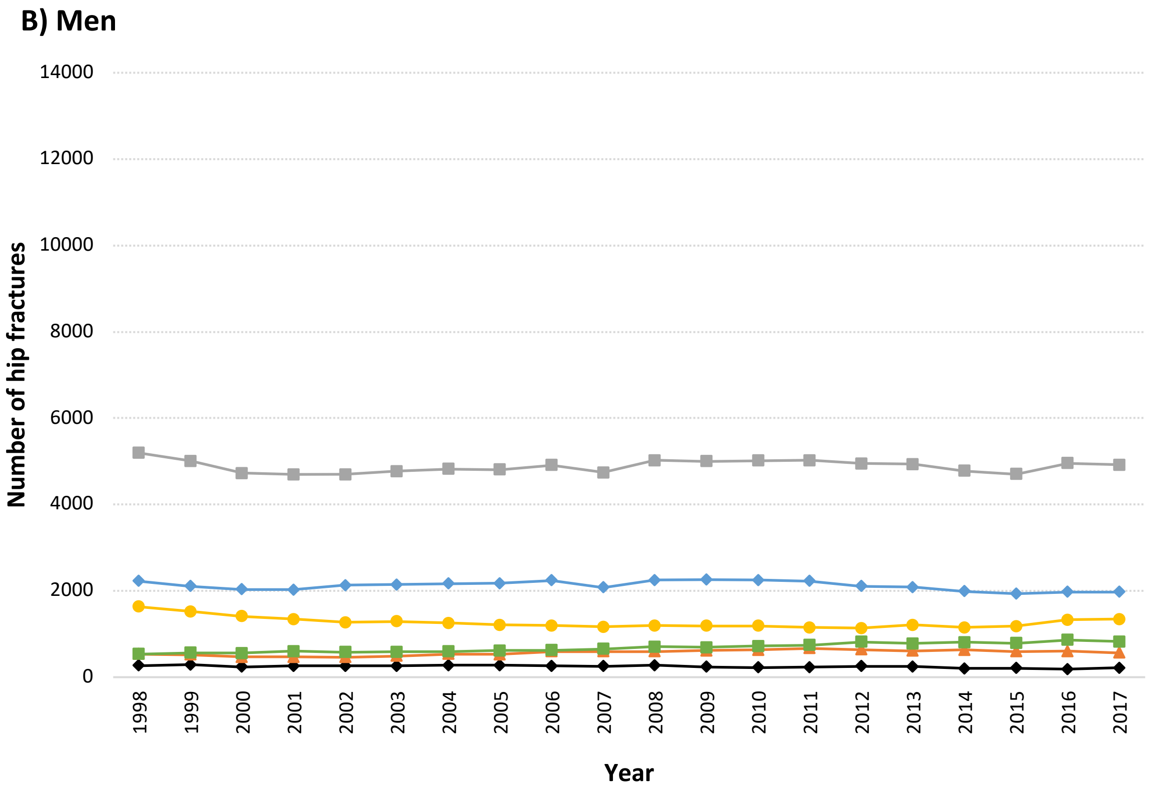

Supplement: Supplementary file 1 — Supplementary file1—Supplementary Fig. S1 Total number of hip fractures from 1998-2017 in Swedish women (A) and in men (B) in total and according to age groups. (PDF 58 kb) [file 223_2022_954_MOESM1_ESM.pdf]
