# Supplementary material for: Trends in Hip Fracture Incidence, Length of Hospital Stay, and 30-Day Mortality in Sweden from 1998–2017: A Nationwide Cohort Study
Source: Calcif Tissue Int. 2022 Feb 15;111(1):21–8. doi: 10.1007/s00223-022-00954-4 (PMC9232476; doi:10.1007/s00223-022-00954-4)
Supplement: Supplementary file 2 — Supplementary file2 (DOCX 24 kb) [file 223_2022_954_MOESM2_ESM.docx]

| Supplementary Table 1. Basal characteristics in 1998 and in 2017 stratified by sex. | | | | |
| --- | --- | --- | --- | --- |
|  | **Men (N=)** | | **Women (N=)** | |
| Year | 1998 | 2017 | 1998 | 2017 |
| Age, years | 79.3±9.6 | 80.3±10.1 | 82.1±8.5 | 82.8±9.5 |
| Income (1000 Swedish krona) | 109±60 | 214±567 | 90±59 | 177±245 |
| Early retirement pension (%) | 4.9 | 3.3 | 2.4 | 2.4 |
| Missing, % | 0.1 | 0.1 | 0.1 | 0.1 |
| Marital status, % |  |  |  |  |
| -Widow/widower | 24.9 | 20.6 | 57.8 | 48.7 |
| -Married | 49.2 | 46.0 | 23.0 | 27.1 |
| -Not married | 14.6 | 15.0 | 9.6 | 8.3 |
| -Divorced | 11.2 | 18.1 | 9.5 | 16.0 |
| Missing, % | 0.1 | 0.1 | 0.1 | 0.1 |
| Length of stay, days (mean ± SD) | 14.3±14.5 | 10.3±7.8 | 15.0±14.4 | 10.2±7.4 |
| Type of fracture and operation |  |  |  |  |
| Collum femoris fracture (%) |  |  |  |  |
| -operated with prosthesis | 2.8 | 32.2 | 5.0 | 32.9 |
| -operated with nails | 19.0 | 7.7 | 18.5 | 7.3 |
| -operated with screws | 17.3 | 7.2 | 16.1 | 6.5 |
| -operated with other technique/unknown | 16.0 | 8.8 | 14.5 | 6.7 |
| Trochanteric fracture (%) |  |  |  |  |
| -operated with intermedullary nail | 1.2 | 16.8 | 1.5 | 20.6 |
| -operated with combined technique | 26.2 | 12.9 | 26.7 | 12.8 |
| -operated with other technique/unknown | 10.5 | 7.6 | 10.7 | 5.4 |
| Subtrochanteric fracture (%) | 7.1 | 6.9 | 7.0 | 7.9 |
| Cause of fracture (%) |  |  |  |  |
| -snow or ice | 5.5 | 4.8 | 2.9 | 2.5 |
| -inside | 67.4 | 52.6 | 72.8 | 59.5 |
| Debut of diagnoses the last year, % |  |  |  |  |
| -myocardial infarction | 1.5 | 0.8 | 1.0 | 0.7 |
| -stroke | 3.7 | 1.9 | 2.6 | 1.5 |
| -angina pectoris | 2.4 | 0.5 | 2.1 | 0.4 |
| -diabetes | 3.0 | 1.8 | 2.6 | 1.1 |
| -kidney failure | 1.0 | 2.3 | 0.4 | 1.1 |
| -obstructive pulmonary disease | 1.4 | 0.9 | 0.8 | 0.7 |
| -alcohol dependency | 3.3 | 2.7 | 3.0 | 2.2 |
| -depression | 1.1 | 0.4 | 1.1 | 0.5 |
| -cancer | 2.5 | 2.8 | 1.5 | 1.8 |
| - dementia | 3.0 | 2.6 | 2.7 | 2.2 |
